# Osseointegration-Related Exosomes for Surface Functionalization of Titanium Implants

**DOI:** 10.34133/bmr.0124

**Published:** 2024-12-20

**Authors:** Boqiong Li, Huanming Chen, Ruiqiang Hang

**Affiliations:** ^1^ Department of Materials Science and Engineering, Jinzhong University, Jinzhong 030619, China.; ^2^ Shanxi Key Laboratory of Biomedical Metal Materials, College of Materials Science and Engineering, Taiyuan University of Technology, Taiyuan 030024, China.

## Abstract

Despite that the clinical application of titanium-based implants has achieved great success, patients’ own diseases and/or unhealthy lifestyle habits often lead to implant failure. Many studies have been carried out to modify titanium implants to promote osseointegration and implant success. Recent studies showed that exosomes, proactively secreted extracellular vesicles by mammalian cells, could selectively target and modulate the functions of recipient cells such as macrophages, nerve cells, endothelial cells, and bone marrow mesenchymal stem cells that are closely involved in implant osseointegration. Accordingly, using exosomes to functionalize titanium implants has been deemed as a novel and effective way to improve their osseointegration ability. Herein, recent advances pertaining to surface functionalization of titanium implants with exosomes are analyzed and discussed, with focus on the role of exosomes in regulating the functions of osseointegration-related cells, and their immobilization strategies as well as resultant impact on osseointegration ability.

## Introduction

The improvement of living standards and medical conditions substantially extend the life expectancy of the population, which increases the incidence rate of dental and orthopedic diseases and thus the need for load-bearing prosthetic replacement [1]. Biomaterials such as titanium and its alloys, stainless steel, Co–Cr–Mo alloys were used as orthopedic implants [2]. Among them, titanium and its alloys show high fatigue strength, favorable biocompatibility, and relatively low modulus, thus are more suitable as implant materials [3]. Despite significant success, they are still actively investigated mainly due to patients’ own diseases such as osteoporosis and diabetes as well as other pathological conditions and/or lifestyle habits, which often lead to implant failure [4]. Many studies have been carried out to modify titanium implants to improve osseointegration ability and implant success [5–8]. Osseointegration is an orchestrated process that occurred at the bone–implant interface. The implant surface characteristics impact the key processes of osseointegration, mainly involving inflammatory reaction, nerve regeneration, angiogenesis, and osteogenesis, and therefore implant fate [9]. Accordingly, surface modification has been deemed as a powerful method to modulate implant osseointegration.

Traditionally, the surface modification mainly focuses on altering the surface topography [10]. For example, sand blasting and acid etching are frequently adopted to create a rough surface on dental implants. Over the past decades, since the topography-based design has reached a high level, further promoting osseointegration from the perspective may be limited. Alternatively, major progress may be expected to occur by surface biochemical modification, through which biomolecules immobilized on the implant surface can directly and efficiently dictate the behavior of osteogenic-related cells. Various proteins, growth factors, and peptides are reported to functionalize titanium implants through physical adsorption or chemical/biological immobilization [11]. For example, Chen and coworkers [12] immobilized a kind of fusion peptide on the titanium surface, which showed favorable osseointegration ability possibly by modulating angiogenic and osteogenic processes.

Recently, exosomes, a kind of extracellular vesicles (EVs), draw increasing attention in biomedical fields including surface functionalization of titanium implants. Compared with previously reported proteins, growth factors, and peptides with relatively simple structure and limited functions, exosomes contain variable proteins, lipids, and nucleic acids, which opens up the possibility of promoting implant osseointegration by modulating multiple processes involved in osseointegration. In this review, we will give a brief overview on the cell biology of exosomes, ranging from their biogenesis and composition to their fate in recipient cells. Then, their biological functions and underlying mechanisms in key processes of osseointegration will be summarized. Then, recent advances in immobilization strategy of exosomes onto titanium surfaces and the resultant biological effects will be discussed in detail.

## Cell Biology of Exosomes

### Exosome biogenesis

In 1981, Trams et al. [13] found small vesicles with membrane structure in the supernatant of cultured sheep reticulocytes in vitro, which was speculated to be a way for cells to excrete waste and was recommended as exosomes. However, in 1996, Raposo et al. [14] found that exosomes secreted by parent cells could modulate the functions of recipient cells. Then, the research on exosomes was rapidly carried out all over the world, and many representative achievements on their biogenesis, composition, and functions were reported.

**Fig. 1.**
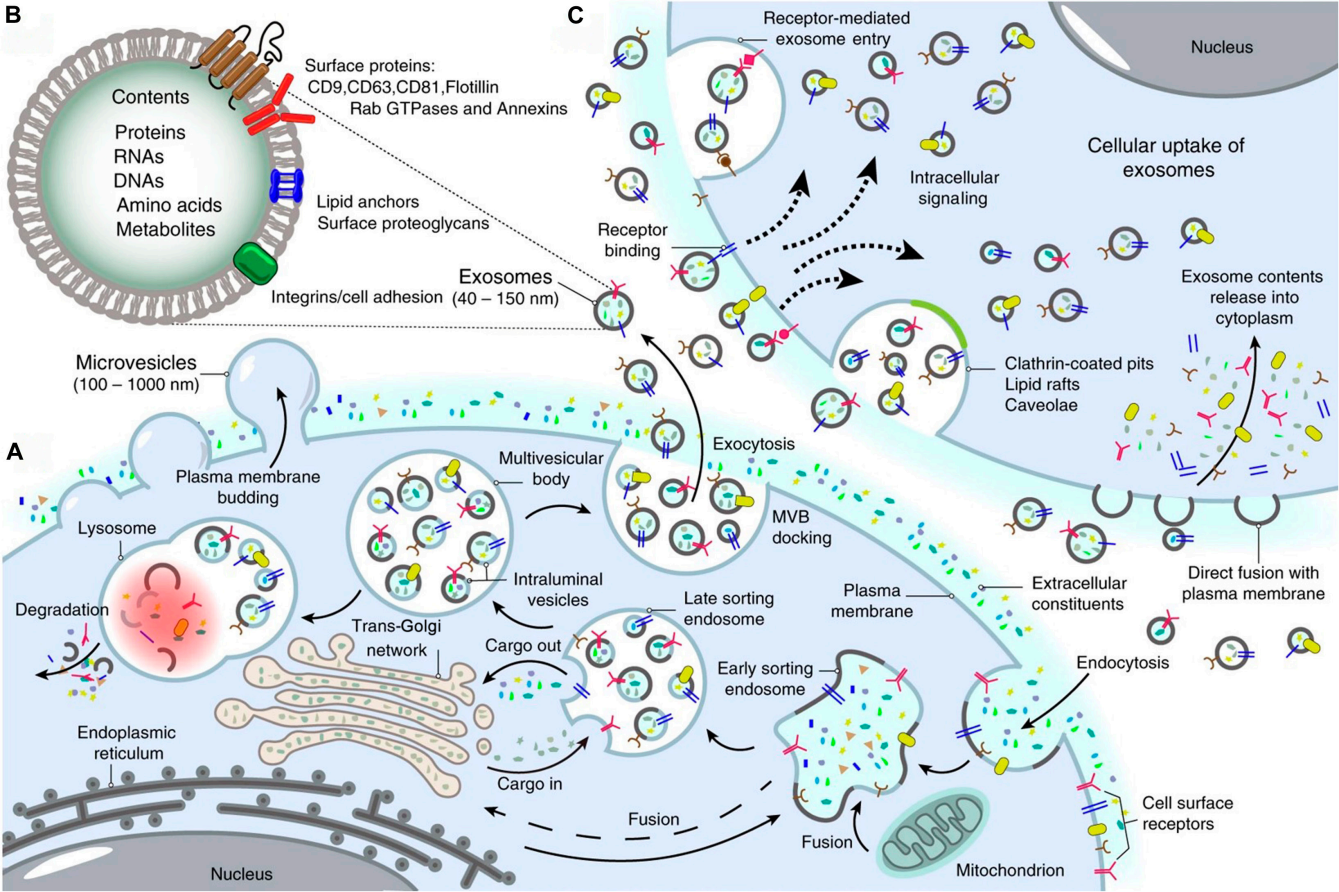
Schematic diagram of cell biology of exosomes. (A) Exosome biogenesis. Exosome biogenesis is a complex and multistep process that involves the formation of intraluminal vesicles (ILVs) within multivesicular bodies (MVBs), followed by the release of these ILVs as exosomes into the extracellular environment. (B) Exosome composition. Exosomes contain a variety type of proteins, nucleic acids, amino acids, and metabolites. (C) Exosome interaction with recipient cells. Exosomes can enter recipient cells through fusion with cell plasma membranes, receptor-mediated entry, clathrin-coated pits, lipid rafts, and so on. Reproduced from [32].

Unlike other EVs such as microvesicles and apoptotic bodies that generate by outward budding of the cell membrane, exosomes are produced through inward budding of endosomal membrane. Although the detailed mechanisms of exosome biogenesis still need to be uncovered, the basic process has been clarified in recent years (Fig. 1A) [15]. Initially, invagination of cell membrane leads to the formation of early endosomes. Next, with the aid of Golgi apparatus, early endosomes mature to late endosomes [also known as multivesicular bodies (MVBs)], during which process intraluminal vesicles (ILVs) are formed through re-invagination of the limiting membrane. The re-invagination is accompanied by recruitment and sorting of cytoplasmic components such as proteins, RNAs, and DNAs into the ILVs. Finally, MVBs fuse with cell membrane and release ILVs, called exosomes.

Several machineries are involved in the formation, intracellular trafficking, and secretion processes of exosomes. Exosomes can be formed through endosomal sorting complex required for transport (ESCRT)-dependent and ESCRT-independent mechanisms [15]. In the ESCRT-dependent process, ESCRT-0 and ESCRT-I capture ubiquitinated cargoes and cluster them on the endosomal membrane, followed by invagination and fission of the membrane at the cluster region motivated by ESCRT-II and ESCRT-III, finally generating MVBs and ILVs [16]. Except for ESCRTs, apoptosis-linked gene 2-interacting protein X (Alix) [17], tumor susceptibility gene 101 (TSG101) [18], and vacuolar protein sorting 4 (Vps4) [18] also participate in the process. Since Stuffers and co-workers [19] found that exosomes could also be generated in the absence of ESCRTs, various ESCRT-independent manners such as ceramide [20] and CD63 [21] pathways are disclosed. After MVB formation, they may be trafficked to lysosomes for degradation or to the plasma membrane for secretion. Although how to balance the degradation and secretion still need to be clarified, the intracellular trafficking mainly involves cytoskeleton (actin and microtubule), molecular motors (dynein and myosin), and molecular switches (small guanosine triphosphatases) [22]. After trafficking to the plasma membrane, the exosome release requires the fusion of MVBs with the membrane, which is regulated by soluble N-ethylmaleimide-sensitive factor attachment protein receptor (SNARE) complex and synaptotagmins [23].

### Exosome composition

Generally, an exosome can be depicted as a lipid bilayer encapsulated vesicle with a variety of transmembrane proteins on its surface and containing various cytoplasmic components of its parent cells such as proteins and nucleic acids (Fig. 1B) [24]. Proteomics analysis showed that the proteins derived from the endosomes, plasma membrane, and cytosol of the parent cells could be identified in their exosomes, while that from other organelles such as nucleus, mitochondria, endoplasmic reticulum, and Golgi apparatus could not be detected [24]. Lipid is the predominant composition of exosome membrane. Compared with parent cell membrane, enrichment of sphingomyelin [25,26], glycosphingolipids [26], phosphatidylserine [26], cholesterol [26], ganglioside GM3 [27], and ceramide [20] was observed in the exosomes. Recent studies also revealed that various nucleic acids, such as mRNA, miRNA, YNRA, rRNA, VTRNA, snRNA, snoRNA, tRNA, and piRNA, could be detected in mammalian cells [28]. The percentage of each RNA within exosomes is quite different from their parent cells, indicating that these contents are incorporated into the exosomes in a precisely controlled manner [28].

Of note, the composition of exosomes derived from specific cells can be varied depending on the extracellular microenvironment. For example, the exosomes derived from human leukemia cells cultured under hypoxic condition highly express miR-210, which can significantly enhance angiogenesis of endothelial cells (ECs) [29]. Another study revealed that tumor cells cultured in acid microenvironment could secrete exosomes enriched with lipids such as sphingomyelin and ganglioside GM3, which in turn increased fusion efficiency with recipient cells [30]. In addition, during the process of differentiation, the mesenchymal stem cells (MSCs) can secrete exosomes with varied composition. The exosomes secreted by MSCs in the late stage of differentiation were enriched with pro-osteogenic microRNAs (miRNAs) [31].

### Exosome interaction with recipient cells

Once secreted into extracellular space from parent cells, the exosomes may target recipient cells through their membrane protein interactions (Fig. 1C). After docking on the cell membrane, exosomes may remain on it and modulate the functions of recipient cells by activating intracellular signaling pathways. Alternatively, the exosomes can release their contents into cytoplasm of recipient cells by direct fusion with the plasma membrane [32]. The exosome can also be ingested by recipient cells through micropinocytosis, phagocytosis, caveolae, clathrin, and lipid raft [15]. Some of the internalized exosomes may target lysosome for degradation, while others may escape the degradation through back fusion with the limiting membrane of MVBs, which results in the release of exosome contents into the cytoplasm of recipient cells.

## Role of Exosomes in Different Stages of Osseointegration

### Inflammatory reaction

Implant osseointegration is an orchestrated process driven by inflammatory reaction, which will affect nerve regeneration, angiogenesis, and osteogenesis, consequently determining implant fate [33–35]. Macrophages (MΦs), the main effector cells of inflammatory reaction, can be quickly recruited to the implant surface after surgery [5,36]. MΦs can switch to different phenotypes in response to extracellular stimuli, and M1 and M2 are 2 extremes. Typically, MΦs showing M1 phenotype secrete a series of inflammatory cytokines [interleukin-1β (IL-1β), tumor necrosis factor α (TNFα), etc.] to induce osteoclastogenesis, resulting in bone resorption, while M2 MΦs contribute to tissue repair such as bone regeneration by secreting osteogenic cytokines [bone morphogenetic protein 2 (BMP2), vascular endothelial growth factor (VEGF), etc.]. In the early phase of inflammation, platelet-derived β-2 microglobulin targets MΦs and polarizes them to pro-inflammatory M1 phenotype by activating the nonclassical transforming growth factor-β receptor (TGF-βR) signaling pathway [37]. The M1 MΦs can combat bacterial infection, phagocytize apoptotic cells, and eliminate bone debris, laying the foundation for tissue repair. Since prolonged M1 phase contributes to fibrosis around implants, timely switch from M1 to M2 is the key to ensure subsequent success of implantation.

**Fig. 2.**
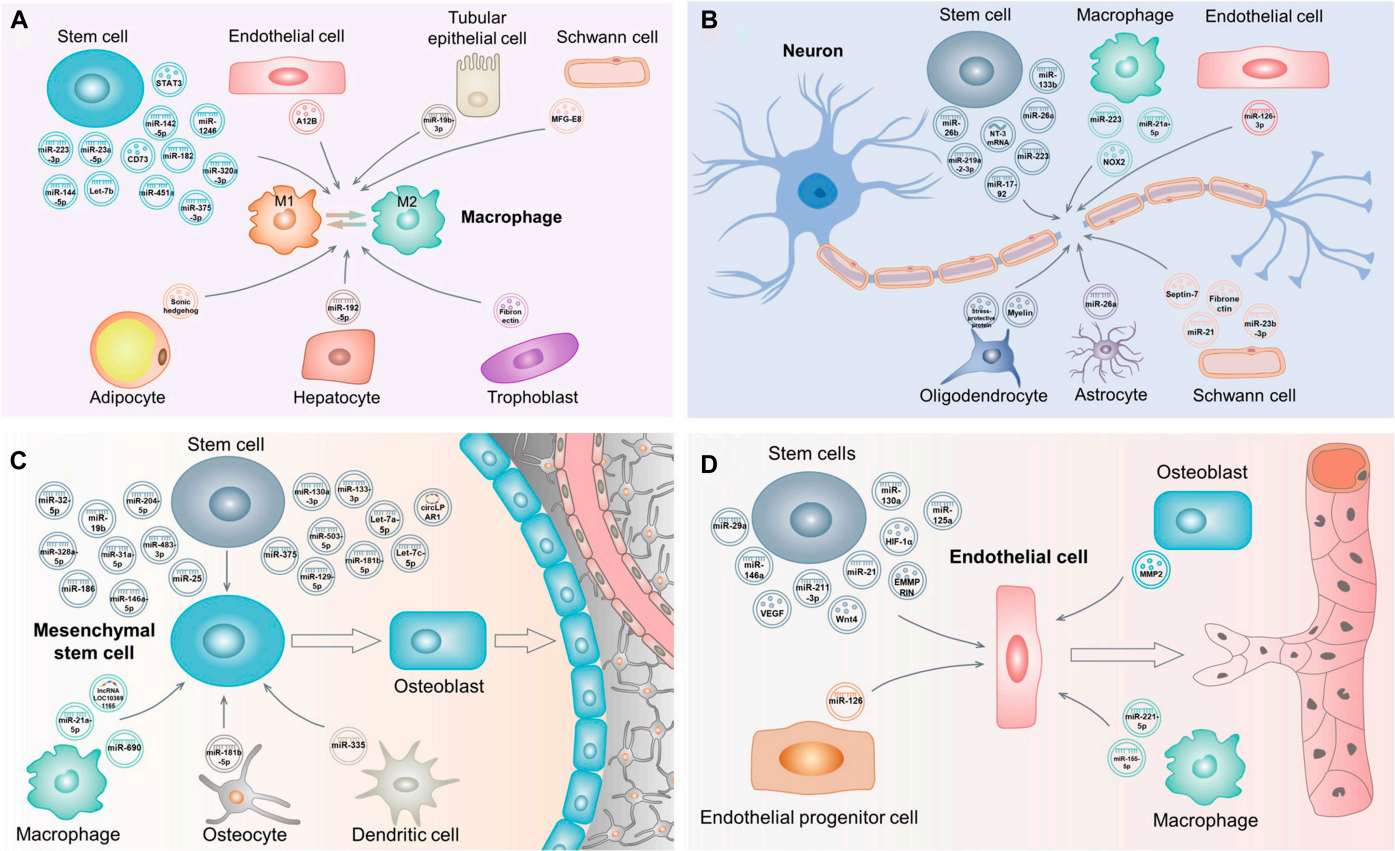
(A) Schematic diagram of macrophage polarization mediated by exosomes derived from different types of cells. (B) Schematic diagram of nerve regeneration mediated by exosomes derived from different types of cells. (C) Schematic diagram of angiogenesis mediated by exosomes derived from different types of cells. (D) Schematic diagram of osteogenesis mediated by exosomes derived from different types of cells. After targeting these cells, the exosomes may release their cargoes to inhibit/promote specific protein synthesis and/or trigger specific signaling pathways to mediate their functions.

In addition to the implant surface physicochemical properties and local delivery of various drugs [33–35,38–41], recent studies also showed that exosomes derived from different types of cells could potently modulate MΦ polarization. Some of the examples are presented in Fig. 2A. Exosomes derived from various stem cells are frequently reported to promote M2 polarization of MΦs, and exosomal miRNAs are the major effectors [42–49]. These miRNAs can target one or more mRNAs of recipient cells to inhibit their translation into proteins. For example, miR-451a enriched in the exosomes derived from adipose-derived stem cells can specifically bind to the 3′ untranslated region (UTR) of migration inhibitory factor (MIF) mRNA of MΦs to down-regulate its expression, which in turn promotes M2 polarization of MΦs [49]. The exosomes of stem cells can also deliver proteins to MΦs. Zhao and coworkers [48] found that signal transducer and activator of transcription (STAT3) enriched in the exosomes derived from adipose stem cells could bind to promoter and enhancer regions of Arg-1 of MΦs and up-regulate its expression and activity, consequently inducing M2 polarization of MΦs. An interesting phenomenon is that extracellular stimuli to stem cells can alter their exosomal constituent, which in turn modulates MΦ polarization. For example, stimulation of mesenchymal stromal cells by lipopolysaccharide (LPS) up-regulated exosomal Let-7b level when compared with that of unstimulated ones, which could polarize MΦs to M2 phenotype by activating the Toll-like receptor 4 (TLR4)/nuclear factor κB (NF-κB)/STAT3/AKT pathway [43]. In addition to various stem cells, exosomes derived from other cells such as ECs, tubular epithelial cells, and Schwann cells can also mediate M2 polarization of MΦs by delivering miRNAs or proteins [50–52]. As previously mentioned, although M2 polarization of MΦs is crucial for the tissue repair, initial M1 polarization is also indispensable. Recent studies showed that the exosomes derived from adipocytes, hepatocytes, and trophoblasts could polarize MΦs to M1 phenotype by delivering sonic hedgehog, miR-192-5p, and fibronectin to MΦs, respectively [53–55]. These exosomes can be incorporated into the titanium implant surface to mediate proper MΦ response to promote osseointegration.

### Nerve regeneration

Bone is innervated by nerve fibers that are connected to the central nervous system. Nerve fibers are distributed in the whole bone tissue, ranging from periosteum and bone marrow to cortical bone and cancellous bone [56]. In nature bone, sensory nerve growth is closely linked to angiogenesis. Nerve growth factor (NGF), secreted by Schwann cells, could stimulate EC proliferation, migration, and angiogenesis by targeting Trk-A on the plasma membrane [57]. NGF could also bind to MΦs, which promote the secretion of VEGF and therefore angiogenesis [57]. In addition, NGF and VEGF secreted by Schwann cells could directly modulate osteogenesis and angiogenesis [58,59]. Denervation compromised bone regeneration and remodeling, and delayed bone fracture healing and osseointegration, mainly by down-regulating osteoblast activity while increasing osteoclast number and up-regulating its activity [56,60–62]. These findings clearly indicate that nerve regeneration is crucial for bone regeneration and osseointegration.

Up to now, few literatures focus on implant osseointegration from the perspective of nerve regeneration. Local injection of NGF to the implant–bone interface and implant surface loading of NGF were proposed to induce peri-implant nerve regeneration [63–65]. In the field of neuroscience, exosome-based strategy to promote nerve regeneration has drawn tremendous attention in recent years. The exosomes derived from a variety of cells can modulate nerve repair and regeneration (Fig. 2B). Among them, nerve-related cell-derived exosomes were intensively investigated. A recent study showed that after insulin-like growth factor-1 stimulation, neural stem cell (NSC)-derived exosomes enriched with miR-219a-2-3p could suppress YY1 expression of PC12 cells, which in turn inhibited neuroinflammation and promoted neuroprotection [66]. Astrocyte-derived exosomes highly expressed with miR-26a could negatively regulate glycogen synthase kinase 3β (GSK3β), phosphatase tensin homolog deletion (PTEN), and brain-derived neurotrophic factor (BDFN) of neurons, thus promoting axon regeneration, neurogenesis, synaptic development, plasticity, and transmission [67]. The exosomes derived from oligodendrocyte were reported to promote axon regeneration mainly by delivering their cargos such as stress-protective proteins and myelin [68]. Schwann cell-derived exosomes enriched with various proteins and miRNAs could also promote nerve repair and regeneration [69–71]. In addition to nerve cell-derived exosomes, the influence of exosomes derived from other cells on nerve regeneration such as stem cells [72–76], ECs [77], and MΦs [78,79] was also reported.

### Angiogenesis

The importance of blood vessels in the formation of the skeleton and in bone repair was documented as early as the 1700s [80]. Bone is a highly vascularized tissue. Blood vessels participate in almost all skeletal functions such as the development, homeostasis, remodeling, repair, and regeneration [81]. Alterations of blood supply to the living bone may result in osteoporosis and osteonecrosis as well as other skeletal diseases [82,83]. Implant osseointegration shares similar process of fracture healing, during which angiogenesis, a process that new blood vessels sprout from a preexisting vascular system, will occur to restore blood supply of compromised skeletal tissue [84–86]. New blood vessels can deliver oxygen, nutrients, cytokines, and osteogenic-related cells to the implant–bone interface to facilitate osteogenesis, which is the key process of osseointegration [34,87].

During the past decade, exosomes released by a variety of cells have been demonstrated to be key mediators of angiogenesis [88]. This review mainly focuses on the exosomes derived from normal cells, because although those from pathological ones such as tumor cells have potent pro-angiogenic ability, their potential risk is not fully understood [89]. Recent studies showed that exosomes derived from various stem cells could facilitate angiogenesis of ECs (Fig. 2C). miRNAs such as miR-29a [90], miR-130a [91], miR-146a [92], miR-125a [93], and miR-21 [94] in the exosomes are the major contents contributing to angiogenesis. For instance, miR-146a can bind to the 3′UTRs of Smad4 and neurofibromin 2 (NF2) mRNAs to inhibit their translation [92]. Smad4 encodes a key intracellular messenger in the TGF-β signaling cascade to inhibit angiogenesis [95], while NF2 is an inhibitor of p21-activated kinase-1 (PAK1) that can up-regulate the expression of VEGF [96], a potent pro-angiogenic cytokine. Accordingly, negative regulation of Smad4 and NF2 by miR-146a can promote angiogenesis. Besides the abovementioned miRNAs, proteins such as Wnt4 [97], VEGF [98], hypoxia-inducible factor-1α (HIF-1α) [99], and extracellular matrix metalloproteinase inducer (EMMPRIN) [100] carried by the exosomes can also mediate angiogenesis of ECs. These proteins exert their pro-angiogenic activity through different pathways. For example, exosome-derived Wnt4 can regulate nuclear translocation of β-catenin of ECs to promote angiogenesis [97], while VEGF can target VEGF receptor (VEGFR) on the membrane of ECs to activate phospholipase Cγ (PLCγ)/protein kinase C (PKC), phosphatidylinositol 3-kinase (PI3K), and mitogen-activated protein kinase (MAPK) signaling pathways, contributing to EC survival, proliferation, and migration [101]. In addition to stem cells, exosomes derived from endothelial progenitor cells, osteoblasts, and MΦs delivering pro-angiogenic factors to ECs were also reported [102–104].

### Osteogenesis

Osteogenesis is defined by a series of events that start with the differentiation of bone marrow mesenchymal stem cells (BMSCs) into osteogenic lineage, followed by proliferation, extracellular matrix (ECM) maturation, and mineralization [105]. BMSCs are adult bone marrow pluripotent stem cells with self-renewal and differentiation capacities. They can differentiate into osteoblasts, fibroblasts, lipoblasts, and chondroblasts upon different microenvironmental stimulation [106]. Whether BMSCs can directionally differentiate into osteoblasts is the key for bone regeneration and osseointegration. For example, fibroblastic differentiation of BMSCs around the implants may result in fibrous encapsulation and implant failure.

It has been reported that various signaling pathways (BMP, WNT, etc.), systemic hormones (parathyroid hormone, estrogens, etc.), local growth factors (TGF-β, VEGF, etc.), as well as epigenetic factors (miroRNAs, noncoding RNAs, DNA methylation, etc.) can govern osteogenic differentiation of BMSCs [107]. Exosome derived from different types of cells regulate osteogenic differentiation of BMSCs mainly through epigenetic factors (Fig. 2D). miRNAs in the exosomes derived from various stem cells, mainly including BMSCs [108–112] and adipose stem cells [113,114], are frequently reported factors to induce osteogenic differentiation of BMSCs. Regarding the possible mechanisms, Zhai and coworkers [109] showed that exosomal miRNAs (miR-146a-5p, miR-503a-5p, miR-129-5p, miR-483-3p, etc.) could activate PI3K/AKT and MAPK signaling pathways to induce osteogenic differentiation of BMSCs by targeting TARF6, FGF2, BMPR, BMP1, and RUNX2. An interesting phenomenon is that the exosomes derived from stem cells in different differentiation stages show altered miRNA profiles [31]. The exosomes derived from parent cells in the late stage of differentiation are enriched with miRNAs (miR-154-5p, miR-10b-5p, miR-18b-5p, miR-152, etc.), which can induce osteogenic differentiation of BMSCs. Moreover, circular RNAs (circRNAs) in the exosomes derived from stem cells were also reported to induce osteogenic differentiation of BMSCs. For example, circular lysophosphatidic acid receptor 1 (circLPAR1) in the exosomes derived from human dental pulp stem cells could bind to miR-31 of BMSCs to eliminate its inhibitory effect on osteogenic differentiation [115].

In addition to stem cells, the exosomes derived from MΦs [116–119], osteocytes [120], and dendritic cells [121] can also induce osteogenic differentiation of BMSCs. Concerning MΦs, conflicting results are reported. For example, Xia and coworkers [116] found that M1 MΦ-derived exosomes rather than M0 and M2 MΦ-derived ones could support osteogenic differentiation of BMSCs, while Wang and coworkers [117] reported that both the exosomes derived from M1 and M2 MΦs could induce osteogenic differentiation of BMSCs because they contained long noncoding RNAs (lncRNAs) (LOC103691165) at similar levels. More detailed works are required to explain the inconsistency. Lv and coworkers [120] found that osteocyte-derived exosomes contained miR-181b-5p, which could directly bind to PTEN to down-regulate its expression, which in turn activated the PI3K/AKT signaling pathway to induce osteogenic differentiation of BMSCs. miR-335 enriched in the exosomes of dendritic cells could target large tongue suppressor kinase 1 (LATS1) and inhibit its expression, consequently inhibiting Hippo signaling, therefore inducing BMSCs differentiated into osteoblasts [121].

## Strategies for Immobilizing Exosomes on Titanium Implant Surfaces and Their Osseointegration Ability

Since exosomes can mediate the functions of osseointegration-related cells, functionalizing titanium implant surfaces with exosomes may be a promising way to promote osseointegration. Currently, immobilization strategy of exosomes on titanium implant surfaces is still in its premature stage. It can be generally divided into 4 approaches, namely, physical adsorption, chemical/biological immobilization, TiO_2_ nanotube (TNT) loading, and hydrogel encapsulation (Fig. S1).

### Physical adsorption

**Fig. 3.**
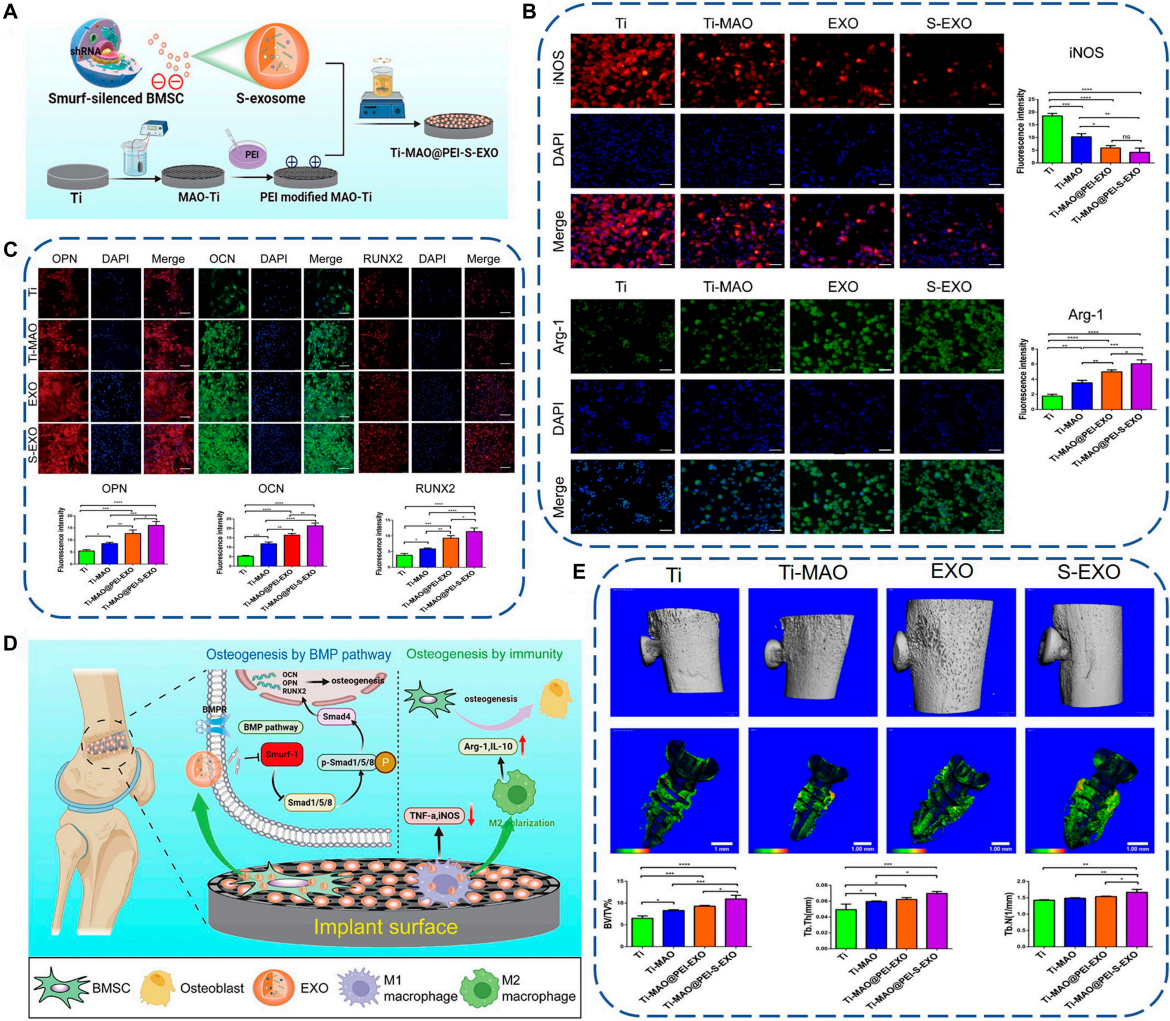
(A) Schematic diagram of preparing exosome-functionalized titanium implant by physical adsorption. (B) Immunofluorescence staining images and quantitative results of M1 marker (iNOS) and M2 marker (Arg-1) of macrophages after culturing on the exosome-functionalized titanium surface for 3 d. (C) Immunofluorescence staining images and quantitative results of OPN, OCN, and RUNX2 of BMSCs after culturing on the exosome-functionalized titanium surface for 21 d. (D) Proposed mechanism of the exosome-functionalized titanium implant promoting osteointegration. (E) 3D reconstructed images of micro-computed tomography scanning and quantification of new bone around the implant after implantation for 8 weeks. Reproduced from [125] with permission from the American Chemical Society.

Physical adsorption is the major method for the immobilization of exosomes on the surfaces of titanium implants. Since exosomes are negatively charged lipid bilayer nanovesicles, they can adsorb to the uncharged titanium surface through electrostatic force. Antich-Rosselló and coworkers [122] successfully immobilized platelet-derived exosomes on the titanium surface by the drop casting method. Although the method is very simple, it may not produce uniformly dispersed exosomes on the titanium surface. To overcome the drawback, Wang and coworkers [123] directly immersed the titanium plate in the culture medium containing BMSC-derived exosomes. Since electrostatic force between the negatively charged exosomes and the uncharged titanium surface is very weak, endowing the titanium surface with positive charge may enhance the electrostatic interaction. Lan and coworkers [124] used acid etching to endow the titanium surface with positive charge and then immersed it into the culture medium containing BMSC-derived exosomes. As expected, the positive titanium surface adhered more exosomes when compared with that of alkali-etched one with negative charge. Similarly, Xu and coworkers [125] used positively charged polyethyleneimine as a linker to adsorb BMSC-derived exosomes on the titanium surface. The preparation process is illustrated in Fig. 3A. The exosome-functionalized titanium facilitated pro-healing M2 polarization and inhibited pro-inflammatory M1 polarization of MΦs, as manifested by down-regulated inducible nitric oxide synthase (iNOS) expression and up-regulated Arg-1 expression when compared with other groups (Fig. 3B). Furthermore, it could also directly up-regulate the expression of OPN, OCN, and RUNX2 of BMSCs, 3 typical markers of osteogenesis (Fig. 3C). The authors also proposed the mechanism of the exosome-functionalized titanium surface promoting osteogenic differentiation of BMSCs (Fig. 3D). On the one hand, the surface polarized MΦs to M2 phenotype to secrete Arg-1 and IL-10 to induce the osteogenic differentiation. On the other hand, the surface could directly promote osteogenic differentiation of BMSCs by activating the BMP/Smad signaling pathway. In vivo experiments also demonstrated that the exosome-functionalized titanium implant possessed better osteointegration ability when compared with other groups (Fig. 3E).

Although physical adsorption is a simple method to immobilize exosomes on the titanium surface, its shortcomings are also obvious. First, the physical interaction between the titanium surface and exosomes is relatively low, so they may be easily detached from the surface, compromising the therapeutic effect. Second, the release of the exosomes from the titanium surface is relatively fast. A research showed that after 8 d of incubation, the exosomes physically adsorbed on the titanium surface were almost completely released [126], while the time period cannot cover the overall osseointegration process. Third, since the first step of implant osseointegration is bleeding and hemostasis, the surface-immobilized exosomes may be ingested by hemocytes in the blood clot rather than osteogenic-related cells.

### Chemical/biological immobilization

**Fig. 4.**
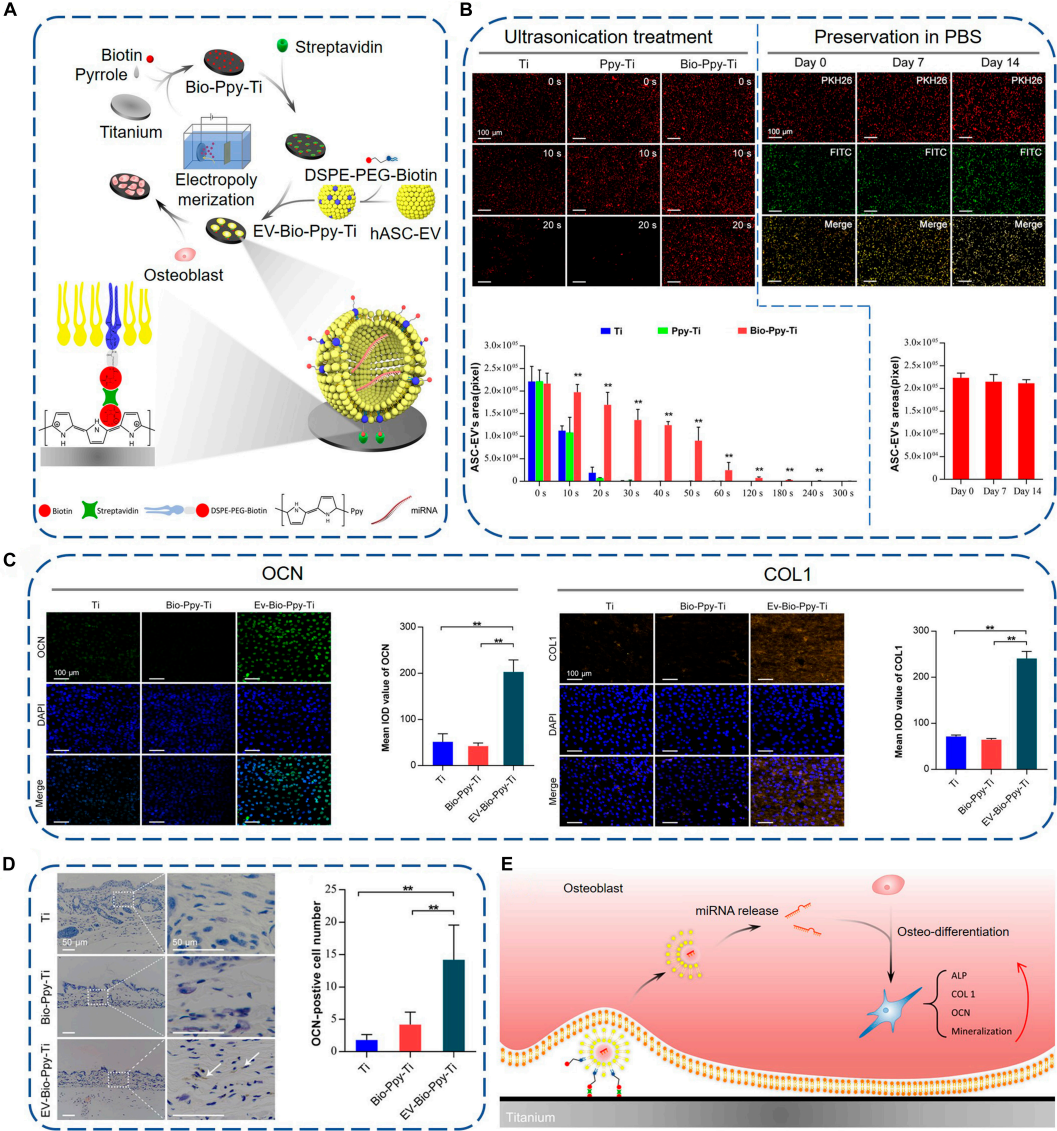
(A) Schematic diagram of preparing exosome-functionalized titanium implant by biological immobilization. (B) Evaluation of the stability of exosome anchoring under ultrasonication treatment (left) and long-term preservation (right). (C) Immunofluorescence staining images of OCN and COL1 as well as their semiquantitative comparison results. (D) Immunohistochemical staining images (left) and quantitative results of OCN-positive cells around the titanium implants. (E) Schematic diagram of exosome-functionalized titanium implants promoting osteogenesis by delivering miRNAs to osteoblasts. Reproduced from [135] with permission from the American Chemical Society.

Compared with physical adsorption, chemical/biological immobilization of exosomes may be more stable. Pansani and coworkers [127] used alkali treatment to produce the nanostructured sodium titanate layer on the titanium surface, followed by plasma activation to form free radicals, which could covalently bind to DMSC-23 cell-derived exosomes. However, they found that the surface treatment could not covalently immobilize fermented papaya fluid-derived exosomes, which was likely that they showed different affinity to the surface due to their different surface composition. Immobilizing exosomes through adsorbed proteins on the titanium surface is another strategy. Fibronectin is a key protein involved in cell adhesion. It contains abundant arginine–glycine–aspartic acid (RGD) sequence, which is the receptor of integrins α5β1, αvβ1, α8β1, αvβ5, αIIβ3, etc. [128], while it is well documented that integrins are highly expressed on the membrane of exosomes [129–131]. Chen and coworkers [132] physically adsorbed fibronectin on the titanium surface and then incubated it in exosome-containing solution. The exosomes were successfully immobilized on the titanium surface through the receptor–ligand interaction. Although the method is relatively simple, the fibronectin may be desorbed from the titanium surface because the interaction between them is weak van der Waals force and electrostatic attraction. Another immobilization strategy may overcome the drawback. Ma and coworkers [133,134] linked titanium-binding peptide (TBP) with CRHSQMTVTSRL (CP05) motif through a linker to fabricate the fusion peptide TBP–CP05. TBP could selectively bind to the titanium surface, while CP05 could bind to tetratransmembrane CD63 on the lipid bilayer of exosomes. Similarly, Chen and coworkers [135] fabricated biotin-doped polypyrrole (Ppy) coating on the titanium surface through electrodeposition, followed by inserting 1,2-distearoyl-sn-glycero-3-phosphoethanolamine-*N*-[biotinyl(polyethylene glycol)] (DSPE-PEG-Biotin) into the phospholipid bilayer of human adipose stem cell-derived exosomes. Then, the titanium and the exosomes were co-incubated with avidin to form the avidin–biotin complex, realizing immobilization of exosomes onto the titanium surface (Fig. 4A). They showed that the immobilization was very stable and reliable, as demonstrated by ultrasonication treatment and long-term preservation in phosphate-buffered saline (PBS) (Fig. 4B). The functionalized titanium implants not only could promote the expression of OCN and COL1 of osteoblasts in vitro (Fig. 4C) but also showed favorable ectopic osteoinductive ability in vivo (Fig. 4D). They also evaluated miRNA expression in the exosomes by gene chips, which found that the exosomes contained multiple miRNAs involved in bone development, such as miR-21, let-7f, miR-10a, and miR-199b. These miRNAs could target osteoblasts to promote osteogenesis (Fig. 4E).

Compared with physical adsorption, chemical/biological immobilization can offer a relatively strong interaction between the titanium surface and exosomes and therefore long preservation period. For example, Chen and coworkers [135] showed that the number of exosomes biologically immobilized onto the titanium surface was 185-fold higher than that of pure titanium and the exosomes remained stable on the titanium surface even after 14 d of preservation in PBS at 4 °C. Nonetheless, as mentioned in the “Physical adsorption” section, the immobilized exosomes may be ingested by hemocytes rather than osteogenic-related cells in in vivo situations.

### TNT loading

**Fig. 5.**
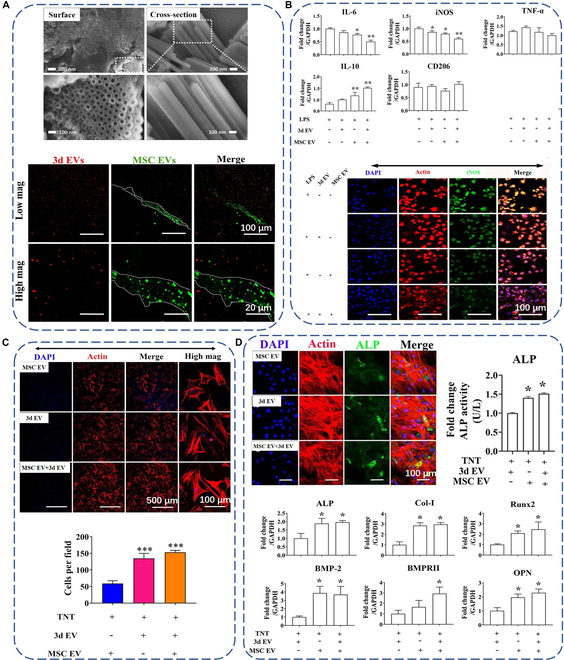
(A) Surface and cross-sectional scanning electron microscopy (SEM) images of TNTs after dopamine coating (top panel) and representative LSCM images of TNT/3d EV/MSC EV hybrid scaffolds (bottom panel). MSC EVs were stained with green PKH67, and 3d EVs were stained with red PKH26. (B) Relative expression of inflammatory-related genes in macrophages cocultured with EV hybrid TNT (top panel) and LSCM images of iNOS expression (bottom panel). (C) Representative LSCM images of hBMSC migration induced by EV hybrid TNT (top panel) and semiquantification results of migrated cells (bottom panel). (D) Qualitative and quantitative ALP expression (top panel) and expression levels of osteogenesis-related genes (bottom panel) of hBMSCs. Reproduced from [137].

TNTs can be fabricated on the titanium surface by anodization, and their dimensions can be precisely controlled by preparation parameters [5]. Their one-end opening geometry renders them favorable drug loading capacity [136]. Zhao and coworkers [137] immersed TNTs coated with polydopamine into the solutions of exosomes derived from MSCs (MSC EV), followed by depositing the chitosan hydrogel layer incorporated with exosomes derived from 3-d osteogenically differentiated MSCs (3d EV) on the TNTs. Figure 5A shows polydopamine-coated TNTs (top panel) and laser scanning confocal microscope (LSCM) images of TNT/3d EV/MSC EV hybrid scaffolds (bottom panel). The TNTs of about 100 nm diameter were successfully fabricated. Before scratching the top chitosan hydrogel layer, only 3d EVs could be observed (in red), while after scratching the layer, the underlying MSC EVs (in green) were exposed, which suggests the successful fabrication of the hybrid scaffolds. The scaffolds could modulate immune response of MΦs, as shown in Fig. 5B. The incorporation of MSC EV and 3d EV could up-regulate the expression of anti-inflammatory genes (IL-10) but could down-regulate the expression of pro-inflammatory genes (IL-6, iNOS, and TNF-α). Immunofluorescence staining of iNOS further indicated that the scaffolds could resolve inflammation. These results clearly indicate that the scaffolds can polarize MΦs to pro-healing M2 phenotype to promote bone regeneration and implant osseointegration. The scaffolds could also directly modulate the functions of human bone marrow-derived MSCs (hBMSCs). As shown in Fig. 5C, incorporation of 3d EVs and/or MSC EVs could promote the migration of hBMSCs, which is critical for the homing of hBMSCs to the implant surface. After homing, the 3d EVs and MSC EVs could also induce osteogenic differentiation of hBMSCs, as manifested by up-regulated alkaline phosphatase (ALP) activity and gene expression related to osteogenesis (Fig. 5D). Using a similar method, Wei and coworkers [138] successfully loaded MΦ-derived exosomes into TNTs. They found that the surface could dramatically up-regulate the expression of early osteogenic differentiation markers, ALP and BMP2 of hBMSCs, possibly because the exosomes could activate autophagy of hBMSCs.

Theoretically, TNTs can realize the sustained release of exosomes within a certain period, but the above 2 works did not measure the release behavior of exosome. In addition, how to control the release kinetics of exosomes from the nanotubes to meet clinical requirements is another concern needing consideration. Furthermore, the selection of nanotube diameter may be a dilemma. Generally, the nanotubes with a large diameter possess few adhesion sites, thus inhibiting cell functions [139], but they can load a large number of exosomes. In contrast, the nanotubes with a small diameter possess plenty of adhesion sites, thus promoting cell functions [140], but they can load relatively few exosomes and even the exosomes cannot be loaded into the nanotubes when their diameter is larger than that of nanotubes. How to balance the dilemma needs further investigation.

### Hydrogel encapsulation

**Fig. 6.**
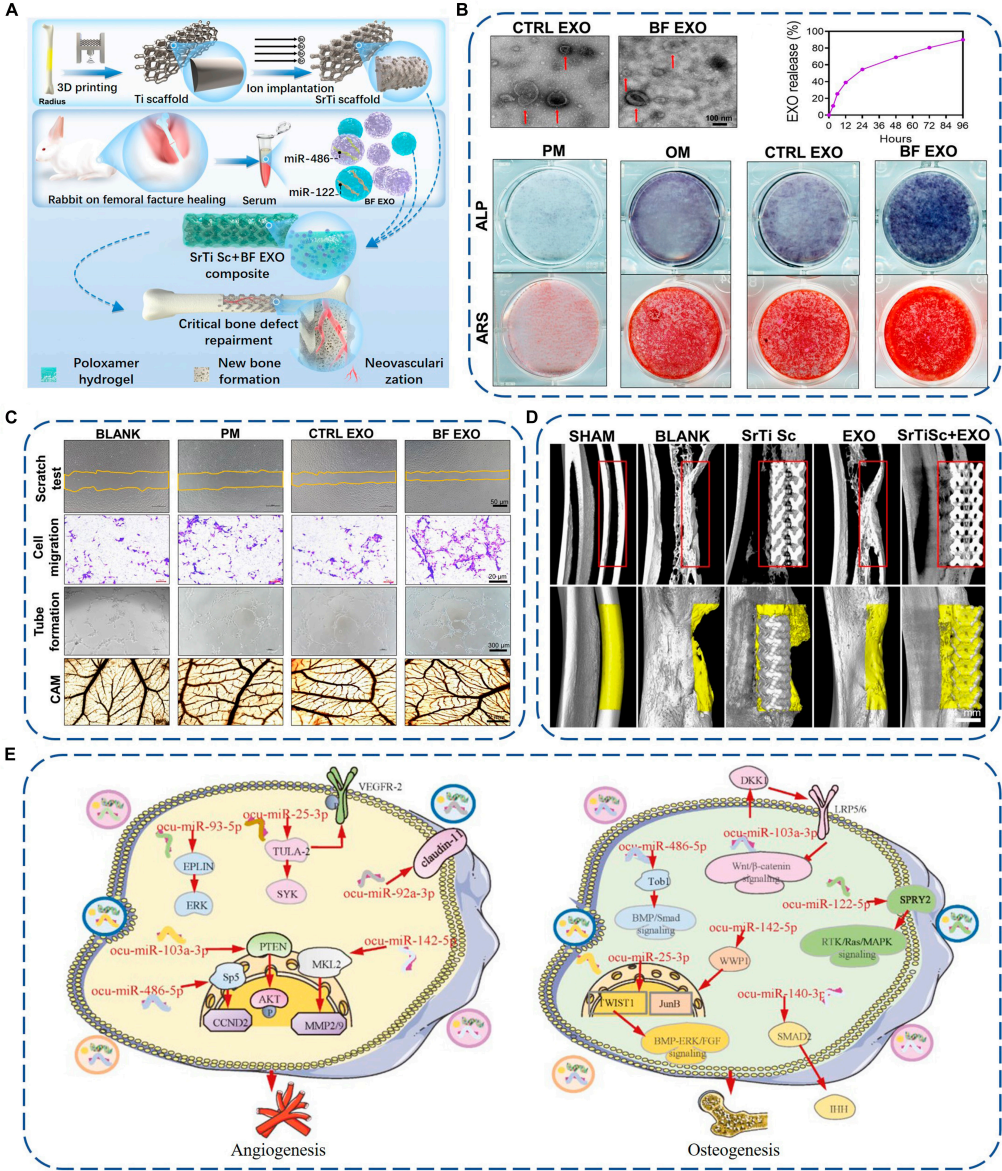
(A) Schematic diagram of preparing exosome-functionalized titanium implant by hydrogel encapsulation. (B) Transmission electron microscopy images of exosomes (top left), exosomes release profile (top right), and images of BMSCs of ALP and alizarin red staining (bottom). (C) Optical images of the scratch test, transwell assay, tube formation, and chicken chorioallantoic membrane (CAM) test of ECs incubated with CTRL EXO or BF EXO. (D) 3D images of the implants and the peri-implant new bone after implantation for 12 weeks. (E) Potential mechanisms of miRNAs in the BF EXO promote angiogenesis and osteogenesis. CTRL EXO: exosomes extracted from the serum of healthy rabbits. BF EXO: exosomes obtained from the serum of rabbits during the period of femoral fracture healing. Reproduced from [146] with permission from the American Chemical Society.

Hydrogels are 3-dimensional systems with hydrophilic polymer chains, which endow them with special properties, including biocompatibility, elasticity, and variable chemical properties [141]. Due to their porous structure, hydrogels have been utilized to realize the controlled release of exosomes. Three main strategies have been reported to encapsulate exosomes into hydrogels, namely, incorporating exosomes into pre-prepared hydrogels through “breathing” technique, mixing exosomes with polymers followed by addition of crosslinkers, and mixing exosomes, polymers, and crosslinkers simultaneously [142]. The progress of hydrogel encapsulation of exosomes for biomedical applications has been summarized by several researchers [141–145]. However, there are only few studies using hydrogels as carriers of exosomes to functionalize titanium implants. Liu and coworkers [146] used poloxamer-based hydrogel to encapsulate serum exosomes. They fabricated titanium scaffold by 3D printing, followed by ion implantation of Sr to generate SrTi scaffold. Then, the scaffold was immersed into the hydrogel containing serum exosomes obtained from the serum of rabbits during the period of femoral fracture healing (BF EXO) to load the exosomes onto the scaffold surface (Fig. 6A). The BF EXO could be slowly released from the hydrogel, which might be responsible for enhanced ALP activity and ECM mineralization of BMSCs (Fig. 6B). In addition, the BF EXO could also promote angiogenesis of ECs, as verified by up-regulated cell migration and tube formation ability in vitro and in vivo (Fig. 6C). Animal experiments showed that the BF EXO-loaded scaffold significantly promoted peri-implant bone regeneration (Fig. 6D). To explain these observations, the authors analyzed miRNA compositions of the BF EXO by high-throughput sequencing. They found that compared with the exosomes extracted from the serum of healthy rabbits (CTRL EXO), BF EXO highly expressed pro-angiogenic and pro-osteogenic miRNAs but lowly expressed anti-angiogenic and anti-osteogenic miRNAs. These miRNAs could target PI3K/AKT/STAT3, CCND2, and IFG/VEGF pathways to promote angiogenesis of ECs, or RTK/Ras/MAPK, BMP/Smad, and WNT/β-catenin pathways to induce osteogenic differentiation of BMSCs (Fig. 6E).

Compared with physical adsorption, chemical/biological immobilization, and TNT loading, hydrogel encapsulation may be an ideal method to load exosomes onto the titanium surface, because it may well control the release kinetics of exosomes by varying polymer type, molecule weight, cross-linking degree, and so on. The well-controlled release of exosomes may ensure that they can be ingested by osteogenic cells rather than other cells such as hemocytes that first arrived at the implant surface upon implantation. From the view point of osteointegration, the ideal hydrogel should be degradable for the following 2 reasons. First, because hydrogels are relatively soft matrix, they may suppress M2 polarization of MΦs [147], angiogenesis of ECs [148], and osteogenic differentiation of BMSCs [149], which are harmful to osseointegration. Second, as substances between host bone and titanium implant, they may hinder their direct contact. How to collaborate the degradation of hydrogels and exosome release to meet clinical requirements needs further exploration.

## Conclusion and Outlook

As emerging intercellular communication media, exosomes have drawn increasing attention in surface functionalization of titanium implants for osseointegration. Upon implantation, the exosomes on the surface of titanium implants can be ingested by osteogenic-related cells and release their contents to modulate the behavior of recipient cells and therefore osseointegration. Two key points for the success of surface functionalization of titanium implants with exosomes are the exosome composition and their immobilization strategy. The composition of the exosomes highly depends on the type of parent cells and microenvironmental stimuli they are exposed to. However, it is still vague that what is the optimal composition of exosomes for osseointegration. In addition, in one type of exosomes, some of the cargoes may facilitate osseointegration, while others may inhibit osseointegration. So, the selection of exosomes is of great importance. Regarding the immobilization strategy, hydrogel encapsulation seems to be better when compared with physical adsorption, chemical/biological immobilization, and TNT loading, but currently, it is difficult to realize the synergy of hydrogel degradation, exosome release, and bone regeneration. Accordingly, further work is required to optimize exosome composition and immobilization strategy to improve outcomes in osseointegration.

Although exosomes are promising in surface functionalization of titanium implants, the following 2 issues should be given due attention to expedite their clinical application. The first is the low yield of exosomes, severely reducing its accessibility. The second is their instability because they contain plenty of active RNAs, lipids, and proteins. To address the first concern, various methods such as lowering pH of the culture medium [150], adding liposomes into the culture medium [151], and culturing the cells in 3D matrix [152] have been used to increase their yield. Regarding the second question, the exosomes or exosome-functionalized implants may be stored at low temperature to maintain exosomal activity [153]. Nonetheless, the 2 issues are not fully addressed up to now. Further work is required to promote the clinical application of exosome-functionalized titanium implants.

## Data Availability

Data sharing is not applicable to this article as no datasets were generated or analyzed during the current study.
