# Supplementary material for: Osseointegration-Related Exosomes for Surface Functionalization of Titanium Implants
Source: Biomater Res. 2024 Dec 20;28:0124. doi: 10.34133/bmr.0124 (PMC11661649; doi:10.34133/bmr.0124)
Supplement: Supplementary 1 — Fig. S1 [file bmr.0124.f1.docx]

**Osseointegration-related exosomes for surface functionalization of titanium implants**

Boqiong Li ^a^, Huanming Chen ^b^, Ruiqiang Hang ^b,^ *

*^a^ Department of Materials Science and Engineering, Jinzhong University，Jinzhong，China. 030619*

*^b^ Shanxi Key Laboratory of Biomedical Metal Materials, College of Materials Science and Engineering, Taiyuan University of Technology, Taiyuan, 030024, China*

* Corresponding author.

E-mail: hangruiqiang@tyut.edu.cn (R.Q. Hang)

**
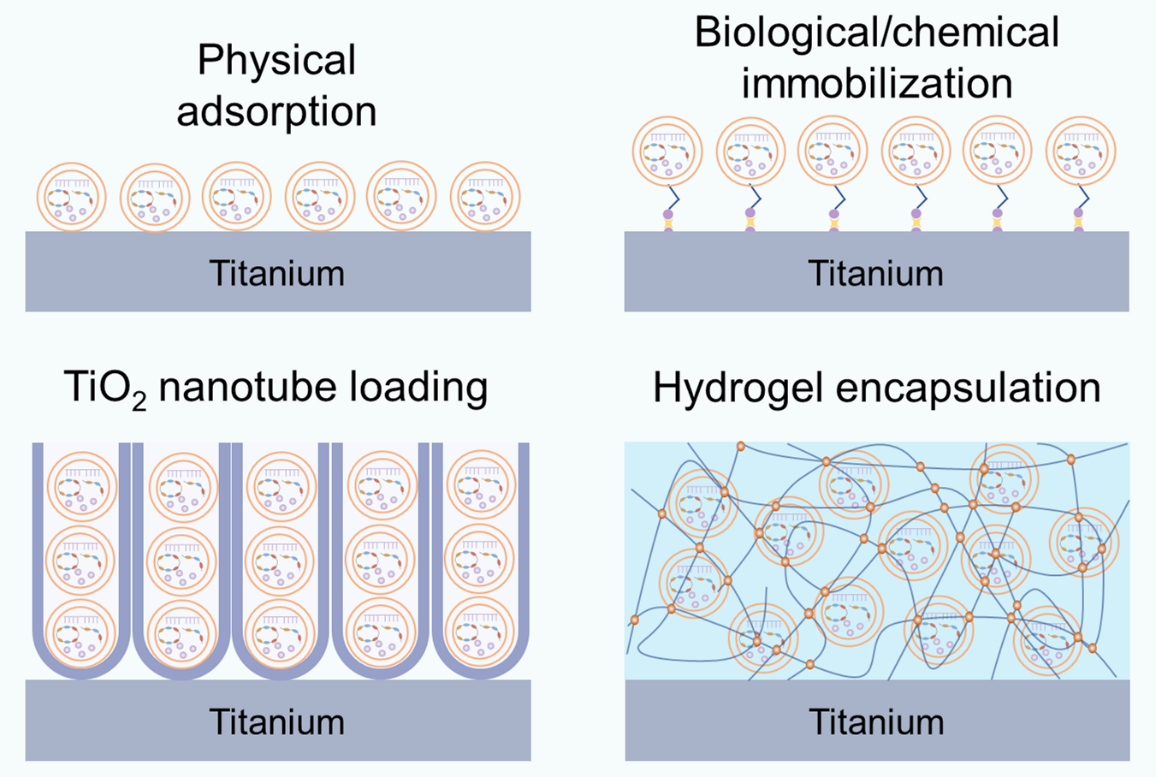
**

**Fig. S1** Schematic diagram of current immobilization strategy of exosomes on titanium implant surfaces
